# Supplementary material for: Personal and Work-Related Burnout Is Associated with Elevated Diastolic Blood Pressure and Diastolic Hypertension among Working Adults in Chile
Source: Int J Environ Res Public Health. 2023 Jan 19;20(3):1899. doi: 10.3390/ijerph20031899 (PMC9915288; doi:10.3390/ijerph20031899)
Supplement: Supplementary file 1 [file ijerph-20-01899-s001.zip › ijerph-2068344-supplementary.pdf]

Supplemental content

Table S1. Association between burnout status and mean systolic blood pressure, diastolic blood pressure, and Hypertension among working adults in Santiago, Chile, with stratification by sex (N = 1,872).

			Mean SBP		Mean DBP		Hypertension			
	N	%	β (95% CI)	P	β (95% CI)	P	n	%	OR (95% CI)	P
Fully adjusted model [a]										
Men	1379	100.0					388	28.5		
No burnout	955	69.3	Ref.	-	Ref.	-	244	25.5	Ref.	-
Only personal burnout	153	11.1	-0.51 (-2.83, 1.81)	0.66	-0.14 (-1.88, 1.60)	0.87	52	34.0	1.25 (0.83, 1.84)	0.28
Only work-related burnout	166	12.0	0.43 (-1.84, 2.70)	0.71	0.43 (-1.27, 2.13)	0.62	68	41.0	1.57 (1.07, 2.28)	0.02
Both	105	7.6	0.95 (-1.78, 3.69)	0.49	2.11 (0.06, 4.15)	0.04	41	39.0	1.65 (1.04, 2.58)	0.03
Women	493	100.0					105	20.6		
No burnout	263	53.3	Ref.	-	Ref.	-	57	21.7	Ref.	-
Only personal burnout	115	23.3	-1.28 (-4.64, 2.09)	0.46	1.15 (-1.11, 3.41)	0.32	27	23.5	1.07 (0.59. 1.91)	0.82
Only work-related burnout	49	9.9	-0.64 (-5.27, 4.00)	0.79	-2.60 (-5.71, 0.52)	0.10	9	18.4	0.82 (0.33, 1.90)	0.66
Both	66	13.4	-2.72 (-6.96, 1.51)	0.21	0.62 (-2.23, 3.46)	0.67	12	18.2	0.68 (0.31. 1.44)	0.33

Abbreviations: β, coefficient; CI, confidence interval; DBP, diastolic blood pressure; OR, odds ratio; Ref., referent group; SBP, systolic blood pressure.

[a] Adjusted for sociodemographic (continuous age, country, and highest education levels), behavioral (BMI, smoking status, alcohol consumption, and physical activity), and occupational characteristics (work sector, work type, and work shift).
